# Psychoeducational Intervention for Sedentary Overweight Adults Who Are Fans of a Football Club: Protocol for a Pragmatic Trial

**DOI:** 10.3390/healthcare14050612

**Published:** 2026-02-28

**Authors:** José A. Jiménez-Chaires, Jeanette M. López-Walle, Abril Cantú-Berrueto, José Tristán, Alejandro García-Mas

**Affiliations:** 1 Faculty of Sports Organization, Autonomous University of Nuevo León, San Nicolás de los Garza 66450, Mexico; abril.cantubrr@uanl.edu.mx (A.C.-B.); jose.tristanrr@uanl.edu.mx (J.T.); alex.garcia@uib.es (A.G.-M.); 2 Department of Psychology, University of the Balearic Islands, 07122 Palma, Spain

**Keywords:** protocol, intervention, psychoeducation, sedentary lifestyle, overweight, autonomous motivation, football fans

## Abstract

**Background**: A sedentary behavior and being overweight represent major public health issues associated with both physical and psychological risks. Based on self-determination theory (SDT), the psychoeducational intervention PsicoFIT—a component of the TIGREFIT program—aims to foster motivation toward physical activity, to promote healthy habits, and to reduce psychological ill-being in sedentary adults who are overweight and are fans of a football club. **Methods**: This protocol corresponds to a longitudinal comparative pragmatic clinical trial, designed in accordance with the recommendations of the SPIRIT Statement. The intervention, preceded by a training program for the coaches involved, will comprise 12 weekly modules delivered in two modalities: (1) face-to-face, through group sessions, and (2) semi face-to-face, through short video capsules hosted on a digital platform. Changes associated with the intervention will be evaluated using hierarchical multiple regression and pre-post comparisons, assessing baseline and post-intervention data within and between the intervention modalities. Primary outcomes will include changes in healthy lifestyle and burnout as indicators of well-being and ill-being, respectively. Secondary outcomes will assess basic psychological needs satisfaction and autonomous motivation as potential mediators of these effects, as well as the coach’s controlling interpersonal style as a possible contextual predictor. The modality of participation will be analyzed as a potential moderator of the observed changes. Finally, the acceptability and perceived contribution of the intervention will be explored through a focus group. **Discussion**: PsicoFIT will provide a methodological framework for designing interventions within multicomponent programs aimed at promoting healthy lifestyles and psychological well-being in sedentary adults who are overweight, considering the social context of football fandom and allowing for an exploration of the impact of the face-to-face and semi-face-to-face modalities. Future empirical application of the protocol will help verify its effectiveness, guide adaptations across contexts, and contribute to the development of evidence-based interventions. **Conclusions**: The implementation of PsicoFit will allow for the evaluation of its effectiveness, psychological mechanisms, and delivery modalities, thus guiding future evidence-based interventions in sport.

## 1. Introduction

A sedentary lifestyle and being overweight constitute major public health concerns affecting a substantial proportion of the adult population worldwide [1,2], and these conditions are closely interrelated [3,4]. Sedentary behavior, predominantly characterized by insufficient engagement in physical activity [5,6], contributes to excess body weight [4], which in turn creates barriers to adopting and maintaining an active lifestyle [7,8,9]. This reciprocal relationship often results in a self-perpetuating cycle with detrimental consequences for both physical health [1,5,10] and psychological well-being [11,12,13,14,15,16].

By contrast, regularly engaging in physical activity has been consistently identified as a protective factor for promoting improvements in physical health and for multiple indicators of psychological well-being [5]. However, initiating and sustaining healthy habits remains particularly challenging among sedentary and overweight populations, where a lack of motivation or behavior regulation primarily driven by external factors often predominates [17,18,19,20].

Self-determination theory (SDT) provides a robust theoretical framework for understanding the motivational processes underlying health-related behavioral change [20,21]. SDT conceptualizes motivation along a continuum, ranging from controlled to autonomous regulation, and emphasizes the role of social and contextual factors in facilitating personal growth and well-being through the satisfaction of three basic psychological needs: autonomy (freedom to choose actions, set goals, and make decisions), competence (sense of achievement and effectiveness in interacting with the environment), and relatedness (maintaining meaningful social connections and a sense of belonging to a community).

From an SDT perspective, well-being is conceptualized in eudaimonic terms, reflecting the individuals’ active engagement in personal development, goal pursuit, and the realization of their potential [20]. Well-being therefore encompasses multiple lifestyle-related domains, including physical activity, adequate rest, balanced nutrition, positive interpersonal relationships, and a sense of life purpose [22].

Autonomous motivation plays a central role in the adoption and maintenance of health-promotion behaviors by facilitating sustained engagement and behavioral persistence [20]. Previous research has shown that autonomous motivation is nurtured by the satisfaction of basic psychological needs and is positively associated with indicators of psychological well-being and healthy lifestyles [20,21,23,24].

According to SDT, less autonomous forms of motivation are associated with higher levels of ill-being [20,21]. Burnout, characterized by a reduced sense of accomplishment, devaluation of the activity, and emotional and physical exhaustion [25,26], has been recurrently framed in sports contexts as an indicator of ill-being. Drawing on this conceptualization, these dimensions may also plausibly manifest in individuals seeking to initiate or to maintain physical activity for health and well-being. The evidence suggests that motivation quality is a relevant predictor of changes in burnout, with lower autonomous motivation typically preceding higher levels of burnout [27,28].

SDT further highlights the importance of support from authority figures to foster autonomous motivation [29], as exemplified by coaches in physical activity settings, through the interpersonal style they adopt in interacting with participants. Research shows that controlling interpersonal styles, characterized by pressures to think, feel, or behave in specific ways, and a disregard for the participants’ interests and perspectives negatively impact autonomous motivation by undermining the satisfaction of basic psychological needs [20,21,30,31,32]. The coach’s interpersonal style is therefore a key contextual factor in comprehensive programs targeting physical activity and well-being.

SDT-based interventions have demonstrated effectiveness in promoting healthy behaviors and psychological well-being among adult populations though both face-to-face and remotely delivered formats. These programs typically include motivational support strategies and health education, and have been shown to enhance autonomous motivation, physical activity engagement, and indicators of well-being.

For example, McDonough et al. [33] reported improvements in autonomous motivation, physical activity, and sleep quality following a 12-week remote intervention for inactive adults using exercise videos and guidance on exercise and sedentary behavior risks, combined with strategies to promote an active lifestyle. Similarly, Dean et al. [34] observed increased physical activity and reductions in body weight following a 10-week program for overweight or obese men combining in-person sessions, activity monitoring, and informational and motivational messages.

Taken together, this body of evidence supports the usefulness of SDT-based interventions for promoting physical activity and psychological well-being in adults. In light of this evidence, it appears pertinent to develop and describe theoretically grounded intervention protocols that explicitly operationalize SDT principles and examine their application across different delivery modalities, including face-to-face and remotely formats.

From an applied perspective, football clubs have been identified as potentially promising settings for the development of initiatives aimed at promoting health and well-being among their supporters, mainly due to their capacity to facilitate recruitment and to provide accessible and socially attractive environments for the implementation of intervention programs [35,36,37,38].

Within this context, TIGREFIT [39] was developed as a multicomponent lifestyle program targeting sedentary and overweight supporters of the professional Mexican football club, Club Tigres, integrating physical training, nutritional guidance, and psychoeducational intervention delivered in the face-to-face and semi face-to-face modalities, along with prior coach training aimed at supporting a motivational approach consistent with the program’s conceptual framework.

PsicoFIT constitutes the psychoeducational component of the TIGREFIT program and was designed to promote motivation toward physical activity and psychological well-being, while reducing indicators of ill-being. The intervention is grounded in SDT and in empirical evidence linking the coach’s interpersonal style, satisfaction of basic psychological needs, and autonomous motivation with key psychological outcomes in physical activity contexts. Accordingly, PsicoFIT was conceptually structured to support autonomy, competence, and relatedness through a progressive psychoeducational approach, providing the theoretical basis for both the intervention design and the evaluation plan.

### Research Objectives and Hypotheses

The purpose of this article is to present the PsicoFIT protocol, detailing its design, implementation, and evaluation plan to guide the intervention. This protocol is embedded within a broader study, whose overall goal is to examine changes resulting from a psychoeducational intervention delivered in the face-to-face and semi face-to-face modalities among sedentary, overweight adults who are supporters of a football club, as well as to evaluate the mediating effects of psychological mechanisms, the moderating effects of each participation modality, and the intervention’s acceptability and perceived contribution by the participants.

To guide the development of this protocol, the following specific objectives and corresponding hypotheses were defined, forming part of the comprehensive study design to be examined in subsequent publications once the intervention has been implemented.

Specific Objective 1. To examine the associations between the coach’s controlling interpersonal style, basic psychological need satisfaction, autonomous motivation, healthy lifestyle, and burnout in the face-to-face and semi face-to-face modalities, both at baseline and post-intervention.

**Hypothesis** **1.** *The coach’s controlling interpersonal style will be negatively associated with basic psychological need satisfaction. In turn, basic psychological need satisfaction will be positively associated with autonomous motivation. Autonomous motivation will be positively associated with healthy lifestyle and negatively associated with burnout in both the face-to-face and semi face-to-face modalities, both at baseline and post-intervention.*

Specific Objective 2. To examine changes in the coach’s controlling interpersonal style, basic psychological need satisfaction, autonomous motivation, healthy lifestyle, and burnout both at baseline and after the psychoeducational intervention, and to compare these changes between the face-to-face and semi face-to-face modalities.

**Hypothesis** **2.** *Significant changes in the coach’s controlling interpersonal style, basic psychological need satisfaction, autonomous motivation, healthy lifestyle, and burnout are expected at post-intervention compared to baseline. Additionally, the magnitude of these changes may differ between the face-to-face and semi face-to-face modalities.*

Specific Objective 3a. To evaluate the mediating effect of the satisfaction of basic psychological needs and autonomous motivation between the coach’s controlling interpersonal style and healthy lifestyle (mediating effect 1), as well as between the coach’s controlling interpersonal style and burnout (mediating effect 2) in both the face-to-face and semi face-to-face modalities, at baseline and post-intervention.

**Hypothesis** **3a1.** *Basic psychological need satisfaction and autonomous motivation will mediate the relationship between the coach’s controlling interpersonal style and healthy lifestyle in both the face-to-face and semi face-to-face modalities, at baseline and post-intervention.*

**Hypothesis** **3a2.** *Basic psychological need satisfaction and autonomous motivation will mediate the relationship between the coach’s controlling interpersonal style and burnout in both the face-to-face and semi face-to-face modalities, at baseline and post-intervention.*

Specific Objective 3b. To evaluate the mediating effect of autonomous motivation between basic psychological need satisfaction and healthy lifestyle (mediating effect 3), as well as between basic psychological needs satisfaction and burnout (mediating effect 4), in both the face-to-face and semi face-to-face modalities, at baseline and post-intervention.

**Hypothesis** **3b1.** *Autonomous motivation will mediate the relationship between basic psychological need satisfaction and healthy lifestyle in both the face-to-face and semi face-to-face modalities, at baseline and post-intervention.*

**Hypothesis** **3b2.** *Autonomous motivation will mediate the relationship between basic psychological need satisfaction and burnout in both the face-to-face and semi face-to-face modalities, at baseline and post-intervention.*

Specific Objective 4. To evaluate the moderating effects of the type of participation (face-to-face and semi face-to-face) both at baseline and after the intervention as follows: (a) the relationship between the coach’s controlling interpersonal style and basic psychological need satisfaction (moderating effect 1); (b) the relationship between basic psychological need satisfaction and autonomous motivation (moderating effect 2); (c) the relationship between autonomous motivation and healthy lifestyle (moderating effect 3); and (d) the relationship between autonomous motivation and burnout (moderating effect 4).

**Hypothesis** **4.** *The type of participation (face-to-face and semi face-to-face) will moderate the relationships between the following both at baseline and after the intervention: (a) the coach’s controlling interpersonal style and basic psychological need satisfaction; (b) basic psychological need satisfaction and autonomous motivation; (c) autonomous motivation and healthy lifestyle; and (d) autonomous motivation and burnout.*

Specific Objective 5. To analyze the acceptability of the PsicoFIT intervention and the perceived contribution by participants in terms of motivation toward physical activity, psychological well-being, and psychological ill-being in both the face-to-face and semi face-to-face modalities.

**Hypothesis** **5.** *The psychoeducational intervention will achieve adequate acceptability, and participants will perceive a positive contribution in terms of motivation toward physical activity, psychological well-being, and psychological ill-being in both modalities.*

The development of this protocol will contribute to establishing methodological guidelines that, once examined, may guide future psychoeducational interventions in sports contexts, particularly those targeting sedentary and overweight adults, by providing a framework for the promotion of healthy habits and psychological well-being.

## 2. Materials and Methods

### 2.1. Design

A mixed-methods convergent parallel approach will be adopted [40] within the framework of a longitudinal comparative pragmatic clinical trial [41]. To examine changes and to compare delivery modalities, the participants will be randomly assigned to receive the intervention in either a face-to-face or semi face-to-face modality, with measurements at time 1 (baseline) and time 2 (post-intervention).

Quantitative analyses will include hierarchical multiple regression analyses, pre-post comparisons, and regression-based mediation and moderation analyses [42,43]. Additionally, the acceptability and perceived contribution of the intervention will be explored using a qualitative phenomenological approach [44] through a post-intervention focus group. The findings from the statistical analysis and the results of the phenomenological analysis will be integrated, allowing for a more precise understanding of the intervention’s evaluation, its perceived impact, and the acceptability of each modality.

Although this study does not constitute a controlled trial and does not include a no-intervention control group, to ensure methodological rigor and scientific transparency, it has been designed in accordance with the standard items of the SPIRIT 2013 Statement for clinical trial protocols [45] (see Supplementary Materials S1) and takes as a reference the elements of the Trials Registration Data Set (TRDS) [46] considered by the World Health Organization (WHO). In addition, the description of the intervention has been guided by the Template for Intervention Description and Replication (TIDieR) checklist [47] (see Supplementary Materials S2), with the aim of providing a comprehensive, transparent, and replicable account of the intervention components, delivery procedures, materials, and implementation context.

### 2.2. Participants and Sample Size

#### 2.2.1. Study Setting

The program will be carried out in Nuevo León, Mexico, in collaboration with the Tigres Football Club and the Autonomous University of Nuevo León. The psychoeducational activities will take place at facilities provided by the university (See Supplementary Materials S3).

The choice of the state of Nuevo León as the study setting is justified by its high combined prevalence of overweight and obesity—76.6% in 2022—higher than the national average of 76.2%, ranking first in the country [48,49]; this is taken together with the strong identity of the supporters of Club Tigres, one of the most popular teams in Mexican football [50].

#### 2.2.2. Recruitment

A call for participation will be disseminated through the official channels of Club Tigres (website and social media) and by email to club subscribers, detailing the general characteristics of the program, participation criteria, expected benefits, and additional incentives. Likewise, academic research centers will support the promotion of the program through their communication channels.

An online registration form will be provided, requesting basic information that will serve as a preliminary eligibility assessment. In addition, contact options by phone and by email will be made available to provide information and to resolve any questions. Finally, a follow-up will be conducted with individuals who express their intention to participate in the program.

#### 2.2.3. Inclusion, Exclusion, and Withdrawal Criteria

The inclusion, exclusion, and elimination criteria for this study will be based on those established by the TIGREFIT program [39].

Inclusion Criteria. Men and women aged 35 to 50 years, with a body mass index (BMI) between 28 and 34.9 kg/m^2^, performing less than 150 min of moderate to vigorous physical activity per week, with at least a secondary education level, access to medical health services, and who provide consent to participate in the study and randomization.

Exclusion Criteria. Individuals with uncontrolled medical conditions, use of medications that significantly alter metabolism, diagnosis of severe psychiatric disorders, pregnancy or breastfeeding, recent or chronic musculoskeletal injuries that limit physical activity, participation in other weight loss or physical activity programs, excessive alcohol or psychoactive substance use, difficulties understanding the study language, or lack of time availability to follow the program.

Withdrawal Criteria. Participants who voluntarily withdraw from the program or those who fail to fully complete the evaluation instruments. 

**Figure 1:**
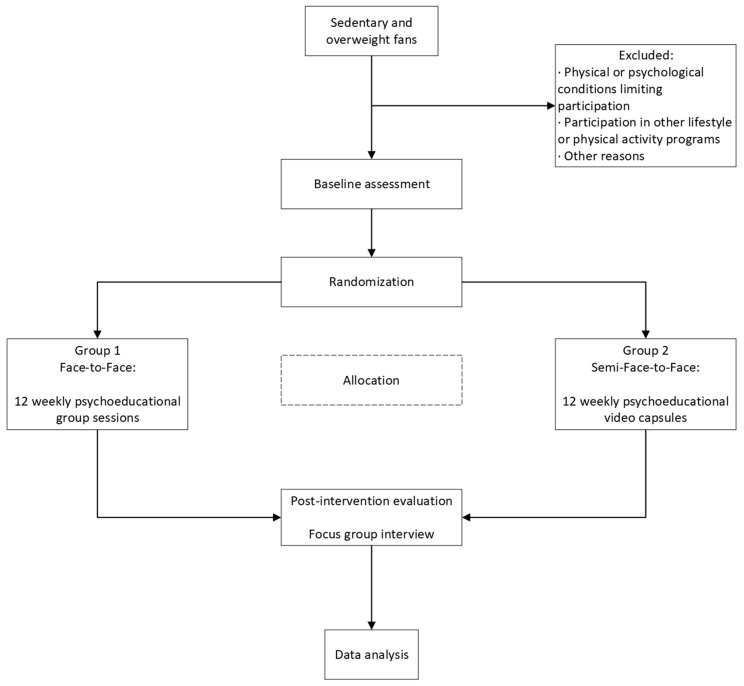
SPIRIT Flow Diagram of the Study Period.

The overall study procedure is presented in Figure 1.

#### 2.2.4. Sample Size and Randomization

The study will employ a non-probabilistic convenience sampling [44], including participants who meet the established inclusion criteria and are available during the recruitment period.

The sample size (*n* = 80) was determined pragmatically and based on operational feasibility, considering the resources and organizational requirements of the intervention, including the facilitators’ capacity, the availability of staff responsible for implementation, the total program duration, and the estimated recruitment period.

The participants will be distributed equally between the two intervention modalities, with 40 participants per modality (20 men and 20 women in each; see Table 1).

Randomization will be conducted using a computer-generated procedure, applying stratification to ensure an equitable distribution between the groups. The randomization will be generated and safeguarded by a researcher independent of the intervention. Once the assignment is generated, each participant will be notified individually.

Blinding of the participants and facilitators is not feasible due to the nature of the intervention delivery format. The face-to-face and semi face-to-face modalities differ in observable aspects, such as the type of interaction, communication channels, and module dynamics, making it impossible to conceal the assigned modality. Nevertheless, participants will not receive information regarding the activities or specific structure of the alternative modality. Likewise, facilitators will necessarily be aware of the format of the modality they deliver.

#### 2.2.5. Participant Retention

Strategies to promote retention in the study will include informational and engagement approaches. An induction session will be conducted to explain the details of the intervention and the schedule of activities, with the aim of reminding the participants of the expected benefits, the importance of their participation, and helping them organize their availability for the program.

Additionally, social incentives linked to Club Tigres will be offered, such as visits to training sessions, match tickets, or interactions with club players.

Participants who accumulate consecutive absences or fail to report viewing the video capsules, whichever the case, will be offered support to continue.

In the case of voluntary withdrawal, the participants will be given the possibility to rejoin the study if feasible; otherwise, they will be considered for future editions of the program.

If a participant leaves the study, their contact information and completed evaluations will be recorded. When possible, they will be asked to complete a survey regarding their experience and reasons for leaving.

### 2.3. Data Collection

#### 2.3.1. Quantitative Evaluation

Quantitative evaluation was conducted using validated and reliable self-report instruments, administered at baseline (time 1) and at the end of the intervention (time 2). Although such measures may be subject to response biases, including social desirability or recall errors, the use of questionnaires with adequate psychometric properties allows for a representative assessment of the variables in the context of the intervention.

Controlling Style of the Coach. The Mexican version of the Controlling Coach Behaviors Scale (CCBS) [51], adapted from the Spanish version [52], will be used. It consists of 15 items on a 7-point Likert scale from 1 (*strongly disagree*) to 7 (*strongly agree*), measuring four controlling motivational strategies in sport: negative conditional regard, intimidation, controlling use of rewards, and excessive personal control. This scale has shown adequate reliability and validity [51,52,53], and has been previously used in exercise and well-being promotion contexts [54].

Psychological Needs Satisfaction. The following instruments, adapted and validated in the Mexican context [55], will be used:

- Perceived Autonomy in Sport Scale (PASS) [56], Spanish version [57], which consists of 10 items on a 7-point Likert scale from 1 (*not at all true*) to 7 (*very true*), evaluating two facets of perceived autonomy: decision-making and volition. The scale has demonstrated adequate reliability and validity [55,56,57].
- Perceived Competence Scale from the Spanish version [57] of the Intrinsic Motivation Inventory (IMI) [58], which is composed of 5 items on a 7-point Likert scale from 1 (*strongly disagree*) to 7 (*strongly agree*), measuring perceived competence in sport. The scale has demonstrated adequate reliability [57,58].
- Need for Relatedness Scale [59], Spanish version [57], which consists of 5 items on a Likert scale from 1 (*strongly disagree*) to 5 (*strongly agree*), measuring perceived acceptance and respect in the sport context. The scale has demonstrated adequate reliability and validity [55,57,59].
- Although these instruments originate from the sports domain, their applicability extends to exercise and well-being contexts, and are recognized by SDT as suitable environments for fostering basic psychological needs [20,21].

Autonomous Motivation. The Sport Motivation Scale II (SMS-II) [60], adapted to the Mexican context [61], will be used. It consists of 18 items on a 7-point Likert scale from 1 (*never*) to 7 (*always*), evaluating six types of motivational regulation. Following previous studies [62,63], autonomous motivation will be measured as a higher-order factor considering the items corresponding to intrinsic, integrated, and identified regulations in coherence with the theoretical framework of this study [20,21]. The scale has shown adequate reliability and validity [61,62,63]. Although this instrument was originally developed in a sports population, its use in exercise and well-being contexts is supported by SDT, which recognizes autonomous motivation as a key determinant of behaviors related to health and well-being, including physical activity [20,21].

Well-Being (Healthy Lifestyle). The “What Is My Lifestyle Like?” Survey developed by the Pan American Health Organization (PAHO) and adapted by the Pontifical Javeriana University [22], will be used. This instrument has also been applied in the Mexican population [64]. It consists of 45 items on a 5-point Likert scale from 1 (*strongly disagree*) to 5 (*strongly agree*), structured into 10 factors: relationship with others, physical activity, rest, nutrition, oral health, sexuality, mobility, substance use, sense of life, and environment. The scale has shown adequate reliability and validity [65], supporting its use in adult populations and its pertinence for application in exercise and well-being contexts.

Ill-Being (Burnout). The Athlete Burnout Questionnaire (ABQ) [25], validated for the Mexican context [26], will be used. It consists of 15 items on a Likert scale from 1 (*almost never*) to 5 (*almost always*), except for items 1 and 14, which are reverse-coded. It evaluates three burnout-related factors: reduced sense of accomplishment, physical and emotional exhaustion, and devaluation of sports practice. The scale has shown adequate reliability and validity [26]. Although this instrument was originally developed for athletic populations, its use in exercise and well-being promotion contexts is considered appropriate in the present study, given that, in addition to PsicoFIT, the program includes other components, such as football fitness, which involves sport-based physical activity, a framework in which burnout dimensions may be relevant for program participants, while acknowledging the limitations inherent in attributing potential changes exclusively to the psychoeducational intervention.

#### 2.3.2. Qualitative Evaluation

A focus group will be conducted with a sample of participants, evenly selected from both modalities, who have provided informed consent. The main topics will be acceptability and perceived contribution in terms of motivation toward physical activity and psychological well-being, with space for suggestions.

#### 2.3.3. Assessment Timeline

Evaluations will be conducted at the following times:

- Time 1. Week 0: What is My Lifestyle Like? Survey.
  Time 1. Week 2: Controlling Coach Behaviors Scale, Perceived Autonomy in Sport Scale, Perceived Competence Scale, Need for Relatedness Scale, Sport Motivation Scale II, and Athlete Burnout Questionnaire. The questionnaires will be administered at this point because they will assess the variables that require the participants to have previously interacted with the program components in order to be appropriately perceived.
- Time 2. Week 13: All instruments and the focus group will be administered.

#### 2.3.4. Data Management

The instruments will be administered in a computer lab using the QuestionPro platform. The collected information will be exported to an Excel database, coded, and prepared for analysis in statistical software.

To safeguard the data, periodic backups will be performed on a secure institutional cloud storage service and a portable external storage device maintained by the research team.

To ensure data quality and accuracy, two researchers will independently enter the information, after which both versions will be compared to detect possible discrepancies or coding errors.

**Table 1:** SPIRIT Schedule for Enrollment, Interventions, and Assessments.

Study Period																
	Enrollment	Allocation		Intervention												Post-Intervention
Time point			w0	w1	w2	w3	w4	w5	w6	w7	w8	w9	w10	w11	w12	w13
Recruitment	X															
Eligibility screening	X															
Informed consent	X															
Allocation		X														
Registration		X														
Interventions																
Intervention A: 12 weeks of face-to-face PsicoFIT with group 1				X	X	X	X	X	X	X	X	X	X	X	X	
Intervention B: 12 weeks of semi face-to-face PsicoFIT with group 2				X	X	X	X	X	X	X	X	X	X	X	X	
Assessments																
Controlling style of the coach					X											X
Psychological needs satisfaction					X											X
Autonomous motivation					X											X
Healthy lifestyle			X													X
Burnout					X											X
Focus group interview																X

### 2.4. TIGREFIT Program

This protocol is part of the TIGREFIT program [39], which includes coach training and three multidisciplinary interventions implemented in parallel. The multicomponent program is offered in two modalities: face-to-face and semi face-to-face. Although some interventions (nutritional and psychoeducational) are delivered virtually in the semi face-to-face modality, the same denomination is maintained, consistent with the structure of the comprehensive program.

#### 2.4.1. Coach Training

Prior to the start of the program, theoretical and practical training will be conducted for the coaches based on the methodology “Teaching with Quality to Generate Well-being in Physical Education and Sport” (ECBEFD) [39], and centered on SDT, high-quality task presentation, and corrective feedback during planning, implementation, and evaluation of the football fitness program.

The training will cover the following topics: (1) motivational climates, basic psychological needs, and quality motivation in promoting and adhering to physical activity; (2) strategies for task presentation, activities, and reinforcement to generate optimal motivational climates and to satisfy basic psychological needs; (3) key criteria for task presentation and feedback to promote well-being; (4) training session planning and practical application; (5) first aid.

Since the first three topics, related to autonomous motivation and basic psychological needs, aim to indirectly influence these variables through the program coaches, the experts responsible for the PsicoFIT intervention will collaborate in delivering this training alongside a multidisciplinary team of experts.

#### 2.4.2. Football Fitness Training

The face-to-face modality includes, on a weekly basis, one hour of recreational training, two two-hour football-based exercise sessions, and 40 min of autonomous recreational physical activity.

The semi face-to-face modality also includes one hour of recreational training (face-to-face), two sessions averaging 24 min of high-intensity interval training (HIIT) through videos hosted through a digital platform, and 40 min of autonomous recreational physical activity per week.

#### 2.4.3. Nutrition Talks

The face-to-face modality consists of a series of weekly 30-min informational talks on nutrition, along with nutritional counseling.

The semi face-to-face modality includes a series of 5-min video capsules on nutrition delivered through a digital platform.

### 2.5. PsicoFIT Intervention

This intervention consists of weekly psychoeducational modules grounded in empirical evidence and structured progressively to support the participants’ understanding, appropriation, and application of the content. The program is designed to promote the development of sustainable health-related behaviors, with a particular emphasis on physical activity, from a motivational perspective that fosters behavioral change through the support of basic psychological needs.

The intervention comprises twelve modules delivered in both the face-to-face and semi face-to-face modalities (Table 2), which follow the same thematic progression but differ in the format of interaction and the depth of activities.

In the face-to-face modality, 12 weekly group sessions lasting approximately 30 min will be conducted. These sessions will include group dynamics, guided exercises implemented in real time, and audiovisual materials aligned with the content of each module.

In the semi face-to-face modality, participants will receive 12 weekly video capsules of approximately five minutes each, delivered through a digital platform. These capsules will consist of structured motivational audiovisual narratives, supported by applied examples, reflection prompts, and key messages aimed at facilitating engagement and the adoption of active behaviors.

In addition to these elements, the intervention will include an indirect component embedded within the football fitness practical session (Module 9), during which motivational principles and functional beliefs will be reinforced through the coaches’ basic psychological need–supportive behaviors during the exercise practice.

#### 2.5.1. Intervention Facilitators

The facilitators responsible for PsicoFIT hold a degree in Psychology and specialized training in Exercise and Sport Psychology. They are in charge of designing, implementing, and supervising the psychoeducational content, as well as guiding the reflection and self-regulation processes throughout the intervention modules. In addition, they provide pre-intervention training for the football fitness coaches, grounded in SDT [20,21], to strengthen the coaches’ support for the participants’ basic psychological needs.

In parallel, within PsicoFIT, the coaches assume an indirect role, adopting a dynamic, flexible, and collaborative approach with an identity oriented toward presenting tasks with quality criteria, promoting an autonomous and supportive climate through activity supervision, providing corrective feedback, and fostering group cohesion [66,67,68,69], acting as a link between intervention strategies, physical practice, and the participants’ motivational processes.

#### 2.5.2. Materials

The PsicoFIT intervention will use structured and standardized psychoeducational materials specifically designed for implementation in both the face-to-face and semi face-to-face modalities.

In the face-to-face modality, audiovisual presentations developed in digital format (e.g., PowerPoint or Canva) will be used, along with motivational and reflective videos and visual support resources (e.g., whiteboard, kraft paper, or other tools intended to facilitate group discussion). Printed materials will also be provided, including worksheets for SMART goal setting and structured formats for the development of functional self-talk. In addition, a guide sheet for coaches will be included, containing specific phrases and guidelines for the application of basic psychological need–supportive behaviors during the football fitness sessions.

In the semi face-to-face modality, structured scripts will be developed for each psychoeducational capsule, integrating audiovisual narratives and graphic resources representative of each module (e.g., animations, explanatory video clips, and dynamic visual supports). Practical activities (such as SMART goal setting and the formulation of self-talk statements) will be presented in an explanatory format within the capsules, encouraging autonomous completion. A coach guide sheet will also be included, providing guidelines for the application of basic psychological need–supportive behaviors during the football fitness sessions.

Additionally, a specific audiovisual presentation on the foundations of SDT will be available and used as supporting material for the training of the football fitness coaches.

All materials will be developed by the research team, ensuring theoretical coherence with the principles of SDT.

#### 2.5.3. Intervention Content

In the first module, the psychological benefits of physical activity will be addressed, emphasizing its positive impact on health, well-being, and various psychological indicators associated with regular engagement in physical activity [70,71,72,73,74].

Subsequently, a module focused on goal planning through the establishment of SMART goals will be developed, with the purpose of promoting specific, measurable, achievable, relevant, and time-bound goals. This module aims to enhance adherence to physical activity and to support the consolidation of sustained behavioral patterns over the long term [75,76,77,78,79].

In addition, a module dedicated to the identification of protective and risk factors associated with physical activity participation will be included. This module seeks to enable the participants to recognize the elements that facilitate or hinder adherence and to develop strategies aimed at strengthening protective factors while reducing the influence of risk factors [80,81,82,83].

In a subsequent module, the basic psychological needs of autonomy, competence, and relatedness will be addressed within the framework of self-determination theory (SDT), with an emphasis on their definition, key characteristics, and the design of strategies aimed at fostering their satisfaction in the context of physical activity and self-care [19,21,29,84,85].

Modules five and six will be specifically oriented toward the promotion of motivation quality, grounded in SDT, with the objective of facilitating behavioral internalization and encouraging more autonomous forms of motivational regulation toward physical activity [19,30,86,87,88].

Complementarily, a module focused on self-talk techniques will be incorporated, aimed at facilitating the regulation of thoughts and emotions related both to physical activity engagement and to everyday situations associated with adherence and personal self-care [89,90,91,92,93].

Subsequently, a module centered on the identification and modification of cognitive distortions will be included, also grounded in rational emotive behavior therapy, fostering a more rational and flexible interpretation of experiences related to physical activity and behavioral change [94,95,96,97,98].

In a complementary manner, a module dedicated to the identification and strengthening of functional beliefs will be developed, based on the principles of rational emotive behavior therapy, with the objective of promoting more adaptive thinking patterns that facilitate emotional and behavioral self-regulation [94,95,98,99,100].

Furthermore, the intervention will include a module focused on the use of behavioral reinforcement strategies, with the purpose of strengthening adaptive behavioral patterns, increasing persistence in physical activity participation, and facilitating long-term behavioral maintenance [70,101,102,103].

Additionally, a module centered on the development of self-control will be integrated, based on principles of social cognitive theory and cognitive behavioral theory, promoting the participants’ capacity to regulate their behavior, to cope with obstacles, and to maintain behavioral changes over time [104,105].

Finally, a module will be incorporated to highlight the importance of adopting a multidisciplinary approach that integrates psychological, nutritional, and physical activity components, with the aim of enhancing the effectiveness, coherence, and sustainability of the intervention [71,106,107,108,109,110].

#### 2.5.4. Procedures

Each session (face-to-face modality) or capsule (semi face-to-face modality) will be delivered following a predefined structure that includes the introduction of the central topic, a theoretically grounded conceptual explanation, applied exemplification within the context of physical activity and daily life, a guided reflection component, and finally, the practical transfer to personal goals and behaviors.

In the face-to-face modality, the facilitators will introduce the module content, develop the corresponding conceptual explanation, and conduct practical and reflective activities aimed at facilitating the participants’ understanding and personal appropriation of the content, as well as its application to specific situations. The session will conclude with the formulation of specific actions intended to implement the learned principles throughout the week in the participants’ daily routines and physical activity practice.

In the semi face-to-face modality, each video capsule will integrate the introduction of the topic through a motivational narrative, the explanation of concepts supported by audiovisual resources, and contextualized examples. Explicit guidelines for reflection and autonomous completion of applied tasks will also be incorporated, promoting the participants’ integration of the weekly content into their physical activity practice and the consolidation of healthy habits.

Module 9 will be implemented in coordination with the football fitness coaches, who will integrate the program principles in a planned manner during the practical session, ensuring coherence between the psychoeducational content and the experiential exercise context.

#### 2.5.5. Setting and Location of Delivery

The face-to-face modality of the program will be delivered in a classroom or enclosed space designated for group educational activities, with adequate lighting, ventilation, and seating arrangements to facilitate interaction and group discussion. The setting will have access to audiovisual equipment (e.g., projector or screen, computer, and internet connection) necessary for the presentation of the psychoeducational content.

The semi face-to-face modality will be implemented through a digital platform that enables the weekly distribution of psychoeducational video capsules. The participants will access the content using their personal devices (e.g., smartphone, tablet, or computer), requiring internet connectivity for viewing.

The practical component in which PsicoFIT Module 9 is embedded will take place in the space designated for the football fitness practice, ensuring appropriate conditions for the safe implementation of physical exercise.

#### 2.5.6. Intervention Tailoring

The content and sequence of the modules are standardized for all participants within each modality. However, consistent with the pragmatic nature of the trial, flexibility will be allowed in the implementation of group dynamics, the contextualization of examples, and the selection of supporting materials or resources, while preserving the predefined conceptual structure and the core motivational mechanisms of the intervention.

#### 2.5.7. Motivational Mechanism of the Intervention

To operationalize SDT, the PsicoFIT intervention was designed to translate its theoretical principles into concrete intervention mechanisms by incorporating motivational strategies aimed at supporting autonomy, competence, and relatedness across all modules and implementation modalities.

Support for Autonomy. Support for autonomy will be promoted throughout the intervention through strategies designed to foster personal decision-making, self-regulation, and the alignment of physical activity with the participants’ individual values and goals.

During the initial phases of the program, the participants will be encouraged to identify and prioritize the exercise-related benefits that are most meaningful to them (Module 1), as well as to set personally relevant and attainable goals, thereby enhancing their sense of choice and personal control over behavior (Module 2).

In a complementary manner, the participants’ capacity to identify and strengthen protective factors, as well as to manage risk factors present in their daily environments, will be fostered, reinforcing perceived control over health- and lifestyle-related decisions (Module 3).

Autonomy will be explicitly addressed as a basic psychological need within the SDT framework, using applied examples related to well-being and physical activity (Module 4), and will be further developed through work on motivational quality, with an emphasis on autonomous motivation and the progressive internalization of motives for exercise engagement (Module 5).

Additionally, autonomy will be incorporated as a central resource for facilitating motivational change, promoting personal choice and self-direction throughout the behavior change process (Module 6). Positive self-talk will be addressed as a strategy for self-guided behavior regulation, directly linked to individual goals and habits (Module 7).

Finally, the program will seek to strengthen autonomy through the use of self-reinforcement strategies (Module 10), the promotion of emotional regulation (Module 11), and the explicit encouragement for the participants to maintain control over their decisions, goals, and habits following the conclusion of the intervention (Module 12).

Support for Competence. Support for competence will be developed by providing a clear structure, opportunities for skill acquisition, and progressive experiences of achievement aimed at strengthening perceptions of personal efficacy. In this regard, the establishment of specific and measurable goals will allow the participants to monitor their progress and to experience greater mastery over their behavior (Module 2), while the analysis of personal and contextual factors will contribute to enhancing their ability to manage the environment effectively (Module 3).

Competence will be explicitly addressed as a basic psychological need through applied examples related to well-being and physical activity (Module 4), and will be further strengthened through the introduction of personally tailored challenges aligned with individual capacities, fostering experiences of success and progressive mastery (Module 6).

The use of positive self-talk applied to personal goals and habits will be oriented toward strengthening perceptions of self-efficacy (Module 7), while the identification and restructuring of cognitive distortions will contribute to consolidating a more functional self-concept and reducing maladaptive interpretations that may undermine perceived competence (Module 8).

Additionally, competence will be addressed indirectly during the football fitness practical session through the coaches’ recognition of achievements and functional behaviors (Module 9). This process will be supported through the use of behavioral reinforcers following the attainment of relevant goals (Module 10), the conceptualization of habit maintenance as a process requiring consistency and perseverance (Module 11), and the recapitulation of the skills and learning outcomes developed throughout the program (Module 12).

Support for Relatedness. Support for relatedness will be promoted by fostering experiences of social connection, interpersonal support, and a sense of belonging within the program, complemented by the symbolic affiliation with the football club as a shared contextual element of social connection.

This need will be explicitly introduced as a central component of well-being and adherence to physical activity (Module 4) and will be strengthened through strategies focused on social support as a key resource for facilitating motivational change (Module 6).

In particular, relatedness will be indirectly promoted through the coaches’ behaviors reflecting closeness, availability, and empathic communication, aimed at facilitating a climate of positive interaction and interpersonal support during the football fitness session (Module 9). Complementarily, social support will be identified as a resource for the development of self-control and perseverance (Module 11) and will be reinforced through an interdisciplinary approach that promotes collaboration among the different areas and professionals involved in the intervention (Module 12).

#### 2.5.8. Intervention Fidelity

To enhance fidelity to SDT-based mechanisms, PsicoFIT incorporates prior coach training grounded in SDT principles, the use of structured psychoeducational materials, and a consistent thematic progression across modules and modes of implementation.

#### 2.5.9. Intervention Adherence

In the face-to-face modality, attendance at sessions will be recorded; to support adherence, reminders and weekly reinforcement messages will be sent to the participants. In the semi face-to-face modality, the participants will report the viewing of the capsules; to encourage adherence, the capsules will be sent with encouraging messages, reinforced with a reminder message, and the participants will submit a screenshot as evidence of capsule viewing.

Only those participants who will have completed both the baseline and post-intervention assessments will be included in the dataset, ensuring that the data reflect actual participation in the program.

#### 2.5.10. Concomitant Care

Restrictions will be established regarding participation in other wellness programs that include psychological, nutritional, or physical activity interventions.

Continuation of any recreational physical activity already practiced by the participants before the study will be allowed, as well as the use of prescribed medications that do not affect variables such as motivation, well-being, or physical activity.

The participants may engage in psychological therapy as long as it does not specifically address motivation for physical activity or psychological well-being using approaches similar to the intervention.

If non-compliance compromising data validity is detected, it will be evaluated whether the participant should be excluded from the final analysis.

#### 2.5.11. Criteria to Interrupt of Modify the Intervention

The intervention will be interrupted for the following situations: if a participant requests it due to discomfort, lack of time, or for any other reason; if serious adverse events directly related to the intervention compromise participant safety; if severe health issues arise that cannot be managed within the protocol, such as a serious illness, injury, or wound.

The research team will have the authority to suspend the intervention under these rules or for any other justified reason.

Modification of the intervention will be considered in the event of moderate adverse events that do not require the interruption of participation but justify a change, such as adjusting the psychoeducational content or reducing the emotional load.

Any modification will be preceded by a thorough evaluation to determine its feasibility and will be submitted to the relevant ethics and research committee before implementation, and informed consent will be obtained from the participants, clearly explaining the reasons and implications of the change. All modifications or interruptions of the protocol will be documented, specifying the reasons, the communication with the participants, and the nature of the corresponding action.

In the event that a participant experiences an adverse effect derived from the intervention, it will be documented in writing and reported to the research ethics committee; psychological attention or appropriate compensation will be offered to remedy any harm.

### 2.6. Data Analysis

#### 2.6.1. Quantitative Analysis

Due to the practical limitations in following up with those participants who do not complete the program, the primary analysis will be conducted using a per-protocol approach [111], considering only those participants with complete data at baseline and at the end of the intervention; missing data will not be imputed.

This approach will allow for evaluating the changes associated with each intervention under real-world implementation conditions, acknowledging the anticipated risk of participant attrition and that the benefits of randomization may not be fully preserved, while reflecting its applicability in a pragmatic context.

All statistical analyses will consider a 95% confidence level (5% error), ensuring transparency and reproducibility.

Preliminary Analysis. Descriptive statistics for central tendency (mean, median), dispersion (standard deviation and variance), and distribution (skewness and kurtosis) will be estimated for each observed variable. Next, normality tests will be conducted, and the reliability of the instruments will be estimated by measuring their internal consistency through Cronbach’s alpha (α) and McDonald’s omega (ω) coefficients. Subsequently, bivariate correlation analyses will be performed to measure the strength and direction of the relationship between the variables.

Associations among motivational and outcome variables. To examine the associations between the coach’s controlling interpersonal style, basic psychological need satisfaction, autonomous motivation, healthy lifestyle, and burnout, hierarchical multiple regression analyses will be conducted. Separate models will be estimated for each intervention modality (face-to-face and semi face-to-face) and for each measurement occasion (baseline and post-intervention), with predictor variables entered in a theoretically driven manner [42]. Effect sizes and confidence intervals will be calculated to complement the statistical significance, and statistical power will be considered when interpreting the results [112].

Intervention-related changes. Changes in the coach’s controlling interpersonal style, basic psychological need satisfaction, autonomous motivation, healthy lifestyle, and burnout from baseline to post-intervention will be examined. Within-group pre-post comparisons will be conducted for each intervention modality (face-to-face and semi-face-to-face). Between-group comparisons will assess whether the magnitude of changes differs between the two modalities [42]. Effect sizes will be calculated to quantify the magnitude of the observed changes, and statistical power will be considered in interpreting the results [113].

Mediation effects. The effects of basic psychological needs satisfaction and autonomous motivation as mediating variables (the mediation effect of basic psychological needs satisfaction and autonomous motivation on the relationship between the coach’s controlling interpersonal style, healthy lifestyle, and burnout, as well as the effect of basic psychological needs satisfaction on the relationship between autonomous motivation, healthy lifestyle, and burnout) will be evaluated using regression-based mediation analyses with bootstrapping procedures to estimate direct and indirect effects following Hayes’s recommendations [43], considering pre- and post-intervention data. The significance of mediation inferred when the confidence interval of the indirect effect does not include zero allows for the assessment of whether the observed relationships are explained directly or indirectly through the proposed mediating variables. Mediation effects will be considered exploratory due to the sample size and the nature of the study.

Moderation Effects. The moderating effects of both intervention modalities will be evaluated using regression-based moderation analyses, following Hayes’s recommendations [43], considering both pre- and post-intervention data. Moderation will be examined by including interaction effects, as evaluated through bootstrapped confidence intervals. Significant interaction effects will indicate that the strength or direction of the relationship between predictors and outcomes differs according to the intervention modality. Moderation effects will be considered exploratory due to the sample size and the nature of the study.

#### 2.6.2. Qualitative Analysis

Acceptability and Perceived Contribution. Participant responses and comments will be analyzed using the constant comparison method [44]. Units of analysis, corresponding to the participants’ opinions, will be systematically compared to identify patterns, differences, and nuances. These units will be categorized using the thematic affinity criterion.

Subsequently, conclusions from the focus group will be compared with the intervention objectives to determine acceptability and perceived contribution, as well as to propose improvements for future implementations.

#### 2.6.3. Integration of Quantitative and Qualitative Findings

Firstly, quantitative and qualitative data will be analyzed independently, following their respective methodologies. Subsequently, the findings will be integrated to address the study’s objectives and hypotheses, providing a more comprehensive understanding of the intervention and its scope.

#### 2.6.4. Data Monitoring

The study will be overseen by the research team, which will verify protocol compliance, data confidentiality, and information safeguarding. Periodic internal meetings will be held to review progress, safety, and protocol adherence.

### 2.7. Expected Primary and Secondary Outcomes

The outcome variables are defined in the following subsections.

#### 2.7.1. Primary Outcomes

- Healthy lifestyle (well-being): Behaviors related to nutrition, physical activity, and other healthy habits will be assessed as indicators of well-being.
- Burnout (ill-being): Physical and emotional exhaustion, sense of accomplishment, and devaluation of sports practice will be evaluated as indicators of ill-being.
- The variable of interest in both cases will be the change after the intervention, calculated as the difference between post-intervention and baseline scores.

#### 2.7.2. Secondary Outcomes

- Basic psychological needs satisfaction: Changes in autonomy, competence, and relatedness will be evaluated as potential mediators of the intervention’s effects.
- Autonomous motivation: Autonomous motivation, composed of intrinsic, identified, and integrated regulation, will be evaluated as a mechanism mediating the effects of the intervention on healthy lifestyle and burnout.
- Controlling interpersonal coaching style: The perception of control and pressure exerted by the coach, including task imposition and reward manipulation, will be assessed as a contextual predictor to understand its influence on the intervention effect sequence.

The metric for all secondary outcomes will be the change in total post-intervention scores relative to baseline.

#### 2.7.3. Acceptability and Perceived Contribution

- Intervention acceptability: The participants’ perception of the PsicoFIT structure, content, and methodology will be evaluated, identifying positively valued aspects and areas requiring adjustments.
- Perceived contribution: The participants’ perceptions of the intervention’s impact on their motivation toward physical activity, adoption of healthy habits, well-being, and management of ill-being will be explored.

This information will complement the quantitative results, providing qualitative evidence on how the participants value the intervention and perceive its contribution to achieving well-being and healthy habits.

### 2.8. Ethical Considerations

This study is part of the protocol titled: “*Programa Integral de Bienestar Incomparables TIGREFIT para población sedentaria y con sobrepeso*” (Comprehensive Unparalleled Well-being Program TIGREFIT for Sedentary and Overweight Population), with registration code CEIFOD 0323 011, approved in its fourth version on 2 August 2023 by the Research Ethics Committee of the Faculty of Sports Organization at the Autonomous University of Nuevo León (CEIFOD; see Supplementary Materials S4). Formal authorization was also obtained from Club Tigres, the institution collaborating in the intervention. All participants were treated respecting the principles established in the Declaration of Helsinki of the World Medical Assembly [113].

Any substantial modification to the protocol will be submitted for review and approval to the ethics committee, and prior notification will be given both to Club Tigres and to the participants.

To ensure voluntary and ethical participation in the program, mandatory informed consent will be obtained, which will be explained and signed by the participants before their inclusion, in compliance with the committee’s guidelines. This process will be carried out by trained members of the research team, who will provide clear and understandable information about the study objectives, the participants’ role, the possible risks and benefits, and the option to withdraw from the study at any time without consequences.

In addition, strict measures will be implemented to protect data confidentiality: participants will be identified using alphanumeric codes, data will be stored in password-protected files and secure servers, and physical documentation will be kept in locked and closed locations. Only authorized personnel will have access to the information, and it will be ensured that the results are reported in aggregate, without identifying the individual participants.

## 3. Discussion

The present protocol outlines a psychoeducational intervention grounded in SDT, aimed at promoting physical activity and enhancing well-being in sedentary adults who are overweight, leveraging the social context generated by football clubs among their supporters as an incentive for participation.

The fact that the participants targeted in the protocol are football fans may introduce a certain behavioral bias [114,115], but it is consistent with the protocol’s framework theory, which is self-determination. Thus, at the beginning of the program, extrinsic reinforcement is needed to ensure—as far as possible—participant adherence [116]; however, once the program is established, the participants will evolve toward autonomy and self-determination, with the secondary objective of reducing the experimental mortality [117,118].

In addition, it is known that there are psychological dynamics in sports teams that help prevent dropout, such as normative and moderate group pressure [119] or increased cohesion [120].

Although it presents inherent methodological constraints, its implementation in both the face-to-face and semi-face-to-face modalities will allow for the analysis of potential changes and intervention-related effects, as well as the acceptability and contribution of each delivery format, and will explore whether the degree of the face-to-face interactions moderate the outcomes [121,122].

This analysis can help in recommending the applicability of the protocol in different areas, since one of the objectives is to provide a framework for the design, implementation, and evaluation of programs aimed at fostering health and well-being, and offers practical guidance to support application and adaptation across different contexts, leading to eventual future replication.

This study presents some important limitations, including the expected rate of participant attrition, which may create bias and reduce the representativeness of the sample, the absence of long-term follow-up, which limits the assessment of the sustainability of behavioral changes, and the use of evaluation instruments, as well as indicators such as burnout, derived from the sports context, whose applicability in sedentary overweight adults should still be considered with caution.

The integration of this protocol within a broader program that includes three interventions and a coach training component implies that observed changes in the study variables may be influenced by the effects of other interventions. While efforts will be made to isolate the specific impact of the present intervention, the results may reflect interactions among different program components, although this problem can also be mitigated to some extent by including it within the same theoretical framework of self-determination, since theoretical coherence could ensure that the strategies, language, and techniques used, as well as the objectives, are consistent, facilitating implementation, acceptance by the participants, and maximizing the potential results [123]. Finally, it should be noted that the results are limited in their generalizability due to the nature of the participants: all of them are football fans, as well as the difference in the “dose” of the intervention between the modalities, which could influence comparative outcomes.

Based on everything discussed and the limitations expressed, it seems clear that the most relevant future development is the empirical application of the protocol in the most efficient way possible, as well as presenting the results obtained, to be replicated for verification and eventual validation as a useful psychoeducational intervention to meet the stated objectives.

## 4. Conclusions

This article presents the PsicoFIT psychoeducational intervention protocol, grounded in Self-Determination Theory, which aims to promote healthy lifestyles and psychological well-being among overweight sedentary adults who are fans of a football club. The detailed description of its theoretical foundation, structure, implementation, and evaluation plan seeks to provide methodological guidance for the development of future interventions integrating psychological and behavioral approaches in sport-related contexts. The future empirical implementation of this protocol will allow for the analysis of its effectiveness, the psychological mechanisms involved, and the usefulness of its different delivery modalities.

## Figures and Tables

**Table 2:** PsicoFIT Intervention.

Module	Objective	Content	Didactic Resources Face-to-Face Modality	Didactic Resources Semi Face-to-Face Modality
1. Psychological Benefits of Physical Activity	Recognize the psychological benefits of physical activity and select the most significant ones	Benefits: self-esteem, self-confidence, enjoyment, safety, stress reduction, mood improvement, concentration, social relationships	Integration dynamics Brainstorming Motivational video	Presentation of the facilitators Illustrative examples Reflection questions Motivational video
2. SMART Goals	Establish specific, measurable, achievable, relevant, and time-bound goals	SMART goals for short, medium, and long term	Development of SMART goals Feedback Group reflection	Guided explanation of SMART methodology Examples of goals
3. Social Support for the Adoption of Healthy Habits	Identify personal, social, and contextual factors that influence habit maintenance	Healthy behaviors, internal and external triggers, risk and protective factors	Guided discussion for functional behavior analysis	Questions about risk and protective factors Examples of behavioral triggers
4. Basic Psychological Needs	Understand psychological needs as the foundation for well-being and exercise adherence	Autonomy, competence, and relatedness	Conceptual presentation Practical examples Motivational video	Conceptual explanation Applied examples
5. Quality Motivation	Differentiate autonomous motivation from controlled motivation and link it to personal goals	Motivational regulations, intrinsic vs. extrinsic motivation, self-determined goals	Experiential activity: “The path to quality motivation”	Applied examples to identify the type of motivation
6. Strategies to Transform Motivation	Apply strategies that promote autonomous and sustainable motivation	Strategies: attention to the present moment, enjoyment of the process, autonomy, social support, personal challenges	Work tables Exchange of strategies Group dialogue	Strategies applied to exercise Final reflection
7. Positive Self-Talk	Promote positive and functional internal dialogue	Types of self-talk: affirmations, reframing, visualization, self-compassion	Construction of self-talk words and phrases Transfer of self-talk to weekly goals	Model phrases presentation Practical guide to incorporate self-talk in habit promotion
8. Cognitive Distortions	Recognize and replace dysfunctional automatic thoughts	All-or-nothing thinking, negative labeling, catastrophizing, comparison, demotivation	Presentation of personal cognitive distortions Group discussion Positive reframing	Examples of belief reframing in exercise practice Final reflection
9. Positive Beliefs	Recognize and reinforce functional beliefs within exercise practice	Motivation, psychological needs, benefits, goals	Content anchoring Integration of positive beliefs through the coach during football fitness session	Content anchoring Integration of positive beliefs through the coach during football fitness session
10. Behavioral Reinforcements	Use internal and social reinforcements to consolidate habits	Reinforcers: positive, negative, social, differential, self-reinforcement	Explanation of types of reinforcers Planning of reinforcers	Conceptual explanation Applied examples Planning of reinforcers
11. Self-Control	Develop skills in planning, emotional regulation, and perseverance	Strategies: routines, records, contingency plan, sustainable habits, social support	Thematic review Exchange of strategies Reflection video	Thematic review Presentation of strategies Reflection video
12. Multidisciplinary Work	Integrate learnings from different disciplines and reinforce commitment to change	Holistic vision, support networks, sustainable lifestyle	Reflection with holistic well-being focus Closing messages and visualization of continuity Farewell and thanks	Reflection with holistic well-being focus Visual summary of intervention Reflection message: “The challenge continues Farewell and thanks

## Data Availability

The dataset generated from this study will not be publicly available in order to protect participant confidentiality and to comply with the conditions of the informed consent. The data collection forms and informed consent template are intended for the exclusive use of the research team, which will be the only party with access to the data. The statistical code used for the analyses may be made available upon reasonable request to the corresponding author, in support of transparency and reproducibility.

## Supplementary Materials

Supplementary Materials S1. SPIRIT Checklist: https://drive.google.com/file/d/1OXTCpg9e0HZn5cW4LQZn3Mag2_UmKyR2/view?usp=sharing (accessed on 18 February 2026); Supplementary Materials S2. TIDieR Checklist for the PsicoFIT Intervention: https://drive.google.com/file/d/1t3Si2BgOnmwHAItv0Gb0dfS1FuP212mO/view?usp=sharing (accessed on 18 February 2026); Supplementary Materials S3. Approval for Use of UANL Facilities: https://drive.google.com/file/d/1Ubr0Uv58lNUEmAklOavEcsgXfxumwFjt/view?usp=drive_link (accessed on 18 February 2026); Supplementary Materials S4. Ethics Committee Approval for the TIGREFIT Program: https://docs.google.com/document/d/11hQPANgIB8zgtB1oJVsk-NUQbVk4G0dI/edit?usp=drive_link&ouid=105837669309396426284&rtpof=true&sd=true (accessed on 20 February 2026).

## Abbreviations

The following abbreviations are used in this manuscript:

SDT	Self-Determination Theory
SPIRIT	Standard Protocol Items: Recommendations for Interventional Trials
TRDS	Trial Registration Data Set
WHO	World Health Organization
TIDieR	Template for Intervention Description and Replication
BMI	Body Mass Index
CCBS	Controlling Coach Behaviors Scale
PASS	Perceived Autonomy in Sport Scale
IMI	Intrinsic Motivation Inventory
SMS-II	Sport Motivation Scale II
PAHO	Pan American Health Organization
ABQ	Athlete Burnout Questionnaire
ECBEFD	Teaching with Quality to Generate Well-being in Physical Education and Sport
HIIT	High-Intensity Interval Training
SEM	Structural Equation Model
SRMR	Standardized Root Mean Square Residual
χ^2^	Chi-square Statistic
Df	Degrees of Freedom
RMSEA	Root Mean Square Error of Approximation
NFI	Normed Fit Index
IFI	Incremental Fit Index
TLI	Tucker–Lewis Index
CFI	Comparative Fit Index
AIC	Akaike Information Criterion
CEIFOD	Research Ethics Committee of the Faculty of Sports Organization

## References

[1] World Health Organization https://www.who.int/es/news-room/fact-sheets/detail/obesity-and-overweight.

[2] Strain T., Flaxman S., Guthold R., Semenova E., Cowan M., Riley L.M., Bull F.C., Stevens G.A., Raheem R.A., Agoudavi K. (2024). National, regional, and global trends in insufficient physical activity among adults from 2000 to 2022: A pooled analysis of 507 population-based surveys with 5·7 million participants. Lancet Glob. Health.

[3] Societat Catalana de Medicina Familiar i Comunitària. http://gestor.camfic.cat/Uploads/ITEM_4335_FULL_2732.pdf.

[4] Vázquez E., Calderón Z., Rico J., Ruvalcaba J., Moreno E. (2019). Sedentarismo alimentación, obesidad, consumo de alcohol y tabaco como factores de riesgo para el desarrollo de diabetes tipo 2. J. Negat. No Posit. Results.

[5] World Health Organization https://www.who.int/es/news-room/fact-sheets/detail/physical-activity.

[6] Crespo-Salgado J.J., Delgado-Martín J.L., Blanco-Iglesias O., Aldecoa-Landesa S. (2015). Basic guidelines for detecting sedentarism and recommendations for physical activity in primary care. Aten. Primaria.

[7] Hamer O., Larkin D., Relph N., Dey P. (2021). Fear-related barriers to physical activity among adults with overweight and obesity: A narrative synthesis scoping review. Obes. Rev..

[8] Atlantis E., Barnes E.H., Ball K. (2008). Weight status and perception barriers to healthy physical activity and diet behavior. Int. J. Obes..

[9] Curran F., Davis M.E., Murphy K., Tersigni N., King A., Ngo N., O’Donoghue G. (2023). Correlates of physical activity and sedentary behavior in adults living with overweight and obesity: A systematic review. Obes. Rev..

[10] Patterson R., McNamara E., Tainio M., De Sá T.H., Smith A.D., Sharp S.J., Edwards P., Woodcock J., Brage S., Wijndaele K. (2018). Sedentary behaviour and risk of all-cause, cardiovascular and cancer mortality, and incident type 2 diabetes: A systematic review and dose response meta-analysis. Eur. J. Epidemiol..

[11] Bozzola E., Barni S., Ficari A., Villani A. (2023). Physical Activity in the COVID-19 Era and Its Impact on Adolescents’ Well-Being. Int. J. Environ. Public Health.

[12] Casanova F., O’Loughlin J., Karageorgiou V., Beaumont1 R.N., Bowden J., Wood A.R., Tyrrell J. (2023). Effects of physical activity and sedentary time on depression, anxiety and well-being: A bidirectional Mendelian randomisation study. BMC Med..

[13] Gray L., Leyland A.H. (2008). Overweight status and psychological well-being in adolescent boys and girls: A multilevel analysis. Eur. J. Public Health.

[14] Hanna F., You E., El-Sherif M. (2023). Editorial: The impact of sedentary behavior and virtual lifestyle on physical and mental wellbeing: Social distancing from healthy living. Front. Public Health.

[15] Mortensen S.R., Grøntved A., Brønd J.C., Ried-Larsen M., Petersen T.L., Jørgensen L.B., Jepsen R., Tang L.H., Skou S.T. (2024). Sedentary activity, sedentary bouts, and patterns of total daily sedentary activity, and their relationship with stress and well-being in individuals with diabetes and prediabetes: The Lolland-Falster Health Study. Ment. Health Phys. Act..

[16] Teno S.C., Silva M.N., Júdice P. (2024). Associations between domains of sedentary behavior, well-being, and quality of life—A cross-sectional study. BMC Public Health.

[17] Gryte O.H., Meland E., Samdal G.B., Fadnes L.T., Vold J.H., Mildestvedt H. (2024). Physical activity and sedentary time after lifestyle interventions at the Norwegian Healthy Life Centres. Prim. Health Care Res. Dev..

[18] Sánchez-Romero E., Ponseti F., Cantallops J., García-Mas A. (2021). The Quantity and Quality of Anxiety Are Mediating Variables between Motivation, Burnout and Fear of Failing in Sport. Int. J. Environ. Res. Public Health.

[19] Teixeira P.J., Carraça E.V., Markland D., Silva M.N., Ryan R.M. (2012). Exercise, physical activity, and self-determination theory: A systematic review. Int. J. Behav. Nutr. Phys. Act..

[20] Ryan R.M., Deci E.L. (2017). Self-Determination Theory: Basic Psychological Needs in Motivation, Development, and Wellness.

[21] Ryan R., Deci E. (2000). Self-Determination Theory and the Facilitation of Intrinsic Motivation, Social Development, and Well-Being. Am. Psychol..

[22] Mora S.L., Múnera F.A. (2015). Evaluación de estilos de vida saludable en la Facultad de Medicina de la Fundación Universitaria de Ciencias de la Salud. Rev. Repert. Med. Cirugía.

[23] Ng J.Y.Y., Ntoumanis N., Thøgersen-Ntoumani C., Deci E.L., Ryan R.M., Duda J.L., Williams G.C. (2012). Self-determination theory applied to health contexts: A meta-analysis. Perspect. Psychol. Sci..

[24] Tang M., Wang D., Guerrien A. (2020). A systematic review and meta-analysis on basic psychological need satisfaction, motivation, and well-being in later life: Contributions of self-determination theory. PsyCh J..

[25] Raedeke T.D., Smith A.L. (2001). Development and preliminary validation of a measure of athlete burnout. J. Sport Exerc. Psychol..

[26] Salazar-González D., Cantú-Berrueto A., López-Walle J. (2019). Cuestionario de Burnout Deportivo (ABQ): Análisis y validación en el deporte mexicano. Cuad. Psicol. Deporte.

[27] Cresswell S.L., Eklund R.C. (2005). Motivation and burnout in professional rugby players. Res. Q. Exerc. Sport.

[28] Lonsdale C., Hodge K. (2011). Temporal ordering of motivational quality and athlete burnout in elite sport. Med. Sci. Sports Exerc..

[29] Deci E.L., Ryan R.M. (2000). The “what” and “why” of goal pursuits: Human needs and the self-determination of behavior. Psychol. Inq..

[30] Deci E.L., Ryan R.M. (1985). Intrinsic Motivation and Self-Determination in Human Behavior.

[31] Bartholomew K.J., Ntoumanis N., Thøgersen-Ntoumani C. (2009). A review of controlling motivational strategies from a self-determination theory perspective: Implications for sports coaches. Int. Rev. Sport Exerc. Psychol..

[32] Mageau G.A., Vallerand R.J. (2003). The coach–athlete relationship: A motivational model. J. Sports Sci..

[33] McDonough D.J., Helgeson M.A., Liu W., Gao Z. (2022). Effects of a remote, YouTube-delivered exercise intervention on young adults’ physical activity, sedentary behavior, and sleep during the COVID-19 pandemic: Randomized controlled trial. J. Sport Health Sci..

[34] Dean D.A., Griffith D.M., McKissic S.A., Cornish E.K., Johnson-Lawrence V. (2018). Men on the Move–Nashville: Feasibility and Acceptability of a Technology-Enhanced Physical Activity Pilot Intervention for Overweight and Obese Middle and Older Age African American Men. Am. J. Men’s Health.

[35] Bunn C., Palmer V., Chng N.R., Andersen E., Gray C.M., Hunt K., Jelsma J.G.M., Morgan H., der Sanden M.N.-V., Pereira H.V. (2023). How European Fans in Training (EuroFIT), a lifestyle change program for men delivered in football clubs, achieved its effect: A mixed methods process evaluation embedded in a randomised controlled trial. BMC Public Health.

[36] Quested E., Kwasnicka D., Thøgersen-Ntoumani C., Gucciardi D.F., Kerr D.A., Hunt K., Robinson S., Morgan P.J., Newton R.U., Gray C. (2018). Protocol for a gender-sensitised weight loss and healthy living programme for overweight and obese men delivered in Australian football league settings (Aussie-FIT): A feasibility and pilot randomised controlled tria. BMJ Open.

[37] Thomsen S.D., Schjødt Garboe F.A., Larsen A.E., Uth J., Krustrup P., Madsen E.E. (2023). Football Fitness as a meaningful driver of occupational identity in female breast cancer survivors. Br. J. Occup. Ther..

[38] Wyke S., Bunn C., Andersen E., Silva M., Van Nassau F., McSkimming P., Kolovos S., Gill J.M.R., Gray C.M., Hunt K. (2019). The effect of a programme to improve men’s sedentary time and physical activity: The european fans in training (EuroFIT) randomised controlled trial. PLoS Med..

[39] Rodríguez J.L.T., Torres A.P.V., López S.G., López-Walle J.M., Nava R.R., Cabanillas M.L., Villa M.A.L., Balsom P.D., Hernández J.d.D.O., Villarreal Y.N.G. (2025). Metodología TIGREFIT: Bienestar y Salud Incomparable.

[40] Creswell J.W., Klassen A.C., Plano Clark V.L., Smith K.C. (2011). Best Practices for Mixed Methods Research in the Health Sciences.

[41] MacPherson H. (2004). Pragmatic clinical trials. Complement. Ther. Med..

[42] Field A. (2024). Discovering Statistics Using IBM SPSS Statistics.

[43] Hayes A.F. (2017). Introduction to Mediation, Moderation, and Conditional Process Analysis: A Regression-Based Approach.

[44] Hernández-Sampieri R., Mendoza Torres C.P. (2018). Metodología de la Investigación: Las Rutas Cuantitativa, Cualitativa y Mixta.

[45] Chan A., Tetzlaff J., Altman D., Laupacis A., Gøtzsche P., Krleža-Jerić K., Hrobjartsson A., Mann H., Dickersin K., Berlin J.A. (2015). Declaración SPIRIT 2013: Definición de los elementos estándares del protocolo de un ensayo clínico. Rev. Panam. Salud Pública.

[46] World Health Organization https://www.who.int/tools/clinical-trials-registry-platform/network/who-data-set.

[47] Hoffmann T.C., Glasziou P.P., Boutron I., Milne R., Perera R., Moher D., Altman D.G., Barbour V., Macdonald H., Johnston M. (2016). Better reporting of interventions: Template for intervention description and replication (TIDieR) checklist and guide. Gesundheitswesen.

[48] Campos-Nonato I., Galván-Valencia O., Hernández-Barrera L., Oviedo-Solís C., Barquera S. (2023). Prevalencia de obesidad y factores de riesgo asociados en adultos mexicanos: Resultados de la Ensanut 2022. Salud Publica.

[49] Shamah-Levy T., Romero-Martínez M., Barrientos-Gutiérrez T., Cuevas-Nasu L., Herrera-González M.P., Alejandre-Mora D.A., Vargas-Olmo J.J., Bautista-Arredondo S., Colchero M.A., Gaona-Pineda E.B. (2023). Encuesta Nacional de Salud y Nutrición Continua 2022 e Indicadores para la Primera Infancia. Resultados de Nuevo León.

[50] MITOFSKY (2025). Afición al Fútbol Soccer México 2025. México. https://www.mitofsky.mx/post/aficion-al-futbol-en-m%C3%A9xico-2025.

[51] Tristán J., Segura G., Magaña H., López-Walle J. (2012). Propiedades psicométricas de la Escala de Conductas Controladoras del Entrenador. Rev. Cienc. Ejerc. FOD.

[52] Castillo I., Fabra P., Marcos D., Gonzalez L., Bartholomew K.J., Fuentes A., Balaguer I. El Estilo Controlador del Entrenador: Análisis de las Propiedades Psicométricas. En Simposio Sobre Adaptación y Validación de Cuestionarios en Psicología del Deporte. Proceedings of the VII Congreso Iberoamericano de Psicología.

[53] Aguirre Gurrola H.B. (2016). Estilo Controlador, Frustración de las Necesidades Psicológicas Básicas y Afectos Negativos en Deportistas Mexicanos. Ph.D. Thesis.

[54] Torregrosa D., Belando N., Alias A., Moreno J.A. (2013). Promoción de la satisfacción con la vida en practicantes de wellness. Cult. Cienc. Deporte.

[55] López-Walle J., Balaguer I., Castillo I., Tristán J. (2012). Autonomy Support, Basic Psychological Needs and Well-Being in Mexican Athletes. Span. J. Psychol..

[56] Reinboth M., Duda J. (2006). Perceived motivational climate, need satisfaction and indices of well-being in team sports: A longitudinal perspective. Psychol. Sport Exerc..

[57] Balaguer I., Castillo I., Duda J. (2008). Apoyo a la autonomía, satisfacción de las necesidades, motivación y bienestar en deportistas de competición: Un análisis de la teoría de la autodeterminación. Rev. Psicol. Deporte.

[58] McAuley E., Duncan T., Tammen V. (1989). Psychometric properties of the intrinsic motivation inventory in a competitive sport setting: A confirmatory factor analysis. Res. Q. Exerc. Sport.

[59] Richer S., Vallerand R. (1998). Construction and validation of the need to belong scale. Eur. Rev. Appl. Psychol..

[60] Pelletier L., Rocchi M., Vallerand R., Deci E., Ryan R. (2013). Validation of the revised sport motivation scale (SMS-II). Psychol. Sport Exerc..

[61] Reyes-Hernández O., Tristán J., López-Walle J.M., García-Mas A. (2021). Team Dynamics Perceptions, Motivation, and Anciety in University Athletes. Sustainability.

[62] Pineda-Espejel H., Alarcón E., López-Ruiz Z., Trejo M., Chávez C. (2016). Propiedades psicométricas de la Escala de Motivación en el Deporte revisada (SMS-II) adaptada al español hablado en México. Rev. Int. Cienc. Deport.

[63] Mosqueda S., López-Walle J. (2022). Climas motivacionales, necesidades psicológicas básicas y motivación en deportistas de una institución privada. Sinéctica Rev. Electrónica Educ..

[64] Bonifacio Ortuño A., Peralya Sánchez J.F. (2018). Proceso de Gestión de Riesgos Psicosociales Para un Comedor Industrial Acorde con la Organización Internacional del Trabajo y la Norma ISO 45001. Bachelor’s Thesis.

[65] Barajas Lizarazo M.A., Gómez Acosta C.A., Escobar Velásquez K.D. (2025). Construct validity and reliability of the ‘Lifestyle (PAHO) in the Adult Population’ questionnaire. Glob. Adv. Integr. Med. Health.

[66] Haerens L., Aelterman N., Van den Berghe L., De Meyer J., Soenens B., Vansteenkiste M. (2013). Observing physical education teachers’ need-supportive interactions in classroom settings. J. Sport Exerc. Psychol..

[67] Mouratidis A., Lens W., Vansteenkiste M. (2010). How you provide corrective feedback makes a difference: The motivating role of communicating in an autonomy-supporting way. J. Sport Exerc. Psychol..

[68] Carpentier J., Mageau G.A. (2016). Predicting sport experience during training: The role of change-oriented feedback in athletes’ motivation, self-confidence and needs satisfaction fluctuations. J. Sport Exerc. Psychol..

[69] Vergara-Torres A.P., Tristán J., López-Walle J.M., González-Gallegos A., Pappous A.S., Tomás I. (2021). Quality of the physical education teacher’s instruction in the perspective of self-determination. Front. Psychol..

[70] Fox K.R. (1999). The influence of physical activity on mental well-being. Public Health Nutr..

[71] Biddle S.J.H., Asare M. (2011). Physical activity and mental health in children and adolescents: A review of reviews. Br. J. Sports Med..

[72] Scully D., Kremer J., Meade M.M., Graham R., Dudgeon K. (1998). Physical exercise and psychological well being: A critical review. Br. J. Sports Med..

[73] Craft L.L., Perna F.M. (2004). The benefits of exercise for the clinically depressed. Prim. Care Companion J. Clin. Psychiatry.

[74] Dishman R.K., O’Connor P.J. (2009). Lessons in exercise neurobiology: The case of endorphins. Ment. Health Phys. Act..

[75] Locke E.A., Latham G.P. (2002). Building a practically useful theory of goal setting and task motivation: A 35-year odyssey. Am. Psychol..

[76] Mann T., De Ridder D., Fujita K. (2008). Self-regulation of health behavior: Social psychological approaches to goal setting and goal striving. Health Psychol..

[77] Gould D., Carson S. (2008). Life skills development through sport: Current status and future directions. Int. Rev. Sport Exerc. Psychol..

[78] Bovend’Eerdt T.J.H., Botell R.E., Wade D.T. (2009). Writing SMART rehabilitation goals and achieving goal attainment scaling: A practical guide. Clin. Rehabil..

[79] Weinberg R., Murphy S. (2013). Goal setting in sport and exercise: Research, theory, and practical applications. The Oxford Handbook of Sport and Performance Psychology.

[80] Dishman R.K., Dishman R.K. (1994). Motivation and adherence in physical activity. Advances in Exercise Adherence.

[81] Sallis J.F., Hovell M.F. (1990). Determinants of exercise behavior. Exerc. Sport Sci. Rev..

[82] Marcus B.H., Forsyth L.H. (2009). Motivation People to Be Physically Active.

[83] Hagger M., Chatzisarantis N. (2008). Self-determination theory and the psychology of exercise. Int. Rev. Sport Exerc. Psychol..

[84] Ntoumanis N. (2005). A prospective study of participation in optional school physical education using a self-determination theory framework. J. Educ. Psychol..

[85] Vallerand R.J., Tenenbaum G., Eklund R. (2007). Intrinsic and extrinsic motivation in sport and physical activity. Handbook of Sport Psychology.

[86] Ryan R.M., Deci E.L., Hagger M.S., Chatzisarantis N.L.D. (2007). Active human nature: Self-Determination Theory and the promotion and maintenance of sport, exercise, and health. Intrinsic Motivation and Self-Determination in Exercise and Sport.

[87] Vallerand R.J., Loisier M. (1999). An integrative analysis of intrinsic and extrinsic motivation in sport. J. Appl. Sport Psychol..

[88] Wilson P.M., Mack D.E., Grattan K.P. (2008). Understanding motivation for exercise: A self-determination theory perspective. Can. Psychol. Psychol. Can..

[89] Gammage K.L., Hardy J., Hall C.R. (2001). A description of self-talk in exercise. Psychol. Sport Exerc..

[90] Hardy J. (2006). Speaking clearly: A critical review of the self-talk literature. Psychol. Sport Exerc..

[91] Hatzigeorgiadis A., Biddle S.J.H. (2008). Negative self-talk during sport performance: Relationships with pre-competition anxiety and goal-performance discrepancies. J. Sport Behav..

[92] Theodorakis Y., Hatzigeorgiadis A., Zourbanos N., Murphy S. (2012). Cognitions: Self-talk and performance. Oxford Handbook of Sport and Performance Psychology.

[93] Tod D., Hardy J., Oliver E. (2011). Effects of self-talk: A systematic review. J. Sport Exerc. Psychol..

[94] Ellis A. (1994). Reason and Emotion in Psychotherapy: Revised and Updated.

[95] Turner M.J., Barker J.B. (2014). Using rational emotive behavior therapy with athletes. J. Appl. Sport Psychol..

[96] Beck A.T. (1979). Cognitive Therapy and the Emotional Disorders.

[97] Burns D.D. (1989). The Feeling Good Handbook.

[98] Beck A.T. (1995). Cognitive Therapy: Basics and Beyond.

[99] Sweeney G.A., Horan J.J. (1982). Separate and combined effects of cue-controlled relaxation and cognitive restructuring in the treatment of musical performance anxiety. J. Couns. Psychol..

[100] Martin J.J., Gill D.L. (1995). The relationships of competitive orientations and self-efficacy to goal importance, thoughts, and performance in high school distance runners. J. Appl. Sport Psychol..

[101] Skinner B.F. (1953). Some contributions of an experimental analysis of behavior to psychology as a whole. Am. Psychol..

[102] Bandura A. (1986). Social Foundations of Thought and Action: A Social Cognitive Theory.

[103] Vansteenkiste M., Deci E.L. (2003). Competitively contingent rewards and intrinsic motivation. J. Personal..

[104] Baumeister R.F., Heatherton T.F. (1996). Self-regulation failure: An overview. Psychol. Inq..

[105] Smith R.E., Smoll F.L., Van Raalte J.L., Brewer B.W. (1996). Psychosocial interventions in youth sport. Exploring Sport and Exercise Psychology.

[106] Pate R.R., Pratt M., Blair S.N., Haskell W.L., Macera C.A., Bouchard C., Wilmore J.H. (1995). Physical activity and public health. JAMA.

[107] Sallis J.F., Owen N. (1998). Physical Activity and Behavioral Medicine.

[108] Kahn E.B., Ramsey L.T., Brownson R.C., Heath G.W., Howze E.H., Powell K.E., Corso P. (2002). The effectiveness of interventions to increase physical activity. Am. J. Prev. Med..

[109] Foster C., Hillsdon M., Thorogood M., Kaur A. (2005). Interventions for promoting physical activity. Cochrane Database Syst. Rev..

[110] Westerterp K.R. (2009). Assessment of physical activity: A critical appraisal. Eur. J. Appl. Physiol..

[111] Argimón J.M., Jiménez J. (2004). Métodos de Invetigación Clínica y Epidemiología.

[112] Cohen J. (2013). Statistical Power Analysis for the Behavioral Sciences.

[113] World Medical Association Declaration of Helsinki: Ethical Principles for Medical Research Involving Human Subjects. 75th WMA General Assembly, Helsinki, Finland, October 2024. https://www.wma.net/es/policies-post/declaracion-de-helsinki-de-la-amm-principios-eticos-para-las-investigaciones-medicas-en-seres-humanos/.

[114] Koo T., Kwon H.H., Shin J., Baeck J. (2025). Is social identity theory enough to cover sports fans’ behavior?: Additional perspective from identity fusion theory. Front. Psychol..

[115] Wann D.L., Branscombe N.R. (1993). Sports fans: Measuring degree of identification with their team. Int. J. Sport Psychol..

[116] Cruz L.G., Roca A. (2017). Efectos del reforzamiento variado y constante sobre la resistencia a la extinción. Rev. Mex. Análisis Conducta.

[117] Carvalho B.J., Verardi C.E.L., Maffei W.S., Monesso C.T. (2019). Reasons that determine the practice of football in athletes of the sub-15 and sub-17 categories of a team from the interior of the state of São Paulo. RBFF-Rev. Bras. Futsal E Futeb..

[118] González-Hernández J., Gómez-López M., Manzano-Sánchez D., Valero-Valenzuela A. (2023). Motivated and without fear of failure: The strength of basic psychological needs in youth Spanish athletes in team sports. J. Hum. Kinet..

[119] Laursen B., Veenstra R. (2021). Toward understanding the functions of peer influence: A summary and synthesis of recent empirical research. J. Res. Adolesc..

[120] Quintana R.N., Palacios J.P. (2025). Cohesión de grupo en educación física: Una revisión sistemática. Horiz. Rev. Investig. Cienc. Educ..

[121] Leung D.K.Y., Wong N.H.L., Yau J.H.Y., Wong F.H.C., Liu T., Kwok W.-W., Wong G.H.Y., Lum T.Y.S. (2023). Hybrid-delivered community psychoeducation for people aged 50 and older: A mixed-method evaluation and lesson learned. Internet Interv..

[122] Sitzmann T., Kraiger K., Stewart D., Wisher R. (2006). The comparative effectiveness of web-based and classroom instruction: A meta-analysis. Personal. Psychol..

[123] Hammerness K. (2006). From coherence in theory to coherence in practice. Teach. Coll. Rec..

